# Schwann cell-derived exosomes containing MFG-E8 modify macrophage/microglial polarization for attenuating inflammation via the SOCS3/STAT3 pathway after spinal cord injury

**DOI:** 10.1038/s41419-023-05607-4

**Published:** 2023-01-30

**Authors:** Jie Ren, Bin Zhu, Guangjin Gu, Wencan Zhang, Junjin Li, Hongda Wang, Min Wang, Xiaomeng Song, Zhijian Wei, Shiqing Feng

**Affiliations:** 1 National Spinal Cord Injury International Cooperation Base, Tianjin Key Laboratory of Spine and Spinal Cord Injury, Department of Orthopedics, Tianjin Medical University General Hospital, Tianjin, China; 2 Department of Orthopedics, Qilu Hospital of Shandong University, Shandong University Centre for Orthopedics, Advanced Medical Research Institute, Shandong University, Jinan, Shandong China; 3 Tianjin Key Laboratory of Lung Cancer Metastasis and Tumor Microenvironment, Tianjin Lung Cancer Institute, Tianjin Medical University General Hospital, Tianjin, China

**Keywords:** Neuroimmunology, Acute inflammation

## Abstract

Macrophage/microglia polarization acts as an important part in regulating inflammatory responses in spinal cord injury (SCI). However, the regulation of inflammation of Schwann cell-derived exosomes (SCDEs) for SCI repair is still unclear. Therefore, we intend to find out the effect of SCDEs on regulating the inflammation related to macrophage polarization during the recovery of SCI. Firstly, the thesis demonstrated that SCDEs could attenuate the LPS- inflammation in BMDMs by suppressing M1 polarization and stimulating M2 polarization. Similarly, SCDEs improved functional recovery of female Wistar rats of the SCI contusion model according to BBB (Basso, Beattie, and Bresnahan) score, electrophysiological assay, and the gait analysis system of CatWalk XT. Moreover, MFG-E8 was verified as the main component of SCDEs to improve the inflammatory response by proteomic sequencing and lentiviral transfection. Improvement of the inflammatory microenvironment also inhibited neuronal apoptosis. The knockout of MFG-E8 in SCs can reverse the anti-inflammatory effects of SCDEs treatment. The SOCS3/STAT3 signaling pathway was identified to participate in upregulating M2 polarization induced by MFG-E8. In conclusion, our findings will enrich the mechanism of SCDEs in repairing SCI and provide potential applications and new insights for the clinical translation of SCDEs treatment for SCI.

## Introduction

Spinal cord injury (SCI) is a devastating disease that can cause severe dysfunction of motor, sensory, and autonomic nerves [1]. Numerous factors are involved in the spread of secondary damage, including trauma-induced ischemia, apoptosis, excitotoxicity, and inflammation [2]. Since most of these secondary injury mechanisms can be initiated and regulated by the immune system, it is obvious that therapies-influenced inflammatory cascades are likely to benefit the functional recovery of SCI [3]. The CNS macrophages mainly include macrophages (bone marrow-derived macrophages, BMDM) and microglia (CNS resident macrophages), which together constitute the main cells of the inflammatory response after SCI [4].

Due to the different inflammatory response signals in the local microenvironment after SCI, CNS macrophages undergo morphological and functional changes, such as the pro-inflammatory macrophages (M1) or anti-inflammatory macrophages (M2) [5, 6]. Increasing evidence holds the view that the pro-inflammatory macrophages (M1) can increase the expression of destructive inflammatory cytokines, including IL-1β and TNF-α, aggravate the damage of host cells and hinder the repair of tissues. On the contrary, M2 macrophages play an anti-inflammatory and tissue-repairing role by removing necrotic tissue debris and releasing protective factors, including IL-4 and IL-10 [7, 8]. Therefore, the targeted intervention of macrophages can be applied for SCI by blocking the recruitment and proliferation of macrophages, inhibiting the activation pathway of the M1 phenotype, and promoting the transformation to M2 macrophages [9, 10].

Exosomes are endoplasmic-derived small vesicles containing diverse biologically active molecules, mainly including nucleic acids and proteins, which play a crucial role in intercellular communication [11, 12]. Be able to cross the blood–brain barrier in the CNS, exosomes can mediate communication between neurons and glial cells, promote neuronal growth, and modulate inflammation responses [13, 14]. Sun et al. reported that exosomes derived from human umbilical cord mesenchymal stem cells could reduce local inflammation and promote nerve repair, thereby improving functional recovery after SCI [15].

In recent years, Schwann cells (SCs) have been shown to play a positive role in nerve repair by promoting axonal dedifferentiation and proliferation and removing myelin sheaths and axonal debris [16, 17]. A growing number of studies have found that Schwann cell-derived exosomes (SCDEs) can promote axonal regeneration and carry proteins closely involved in suppressing inflammation [18, 19]. It is indicated that SCDEs have broad application prospects for SCI. Our previous studies have found that SCDEs can, on the one hand, promote axonal protection by increasing autophagy and reducing apoptosis through EGFR/Akt/mTOR signaling pathway, and on the other hand, increase Toll-like receptor 2 expression in astrocytes and reduce the deposition of chondroitin sulfate proteoglycan, thereby improving functional recovery after SCI [20, 21]. Therefore, the present studies intend to further figure out the effect of SCDEs on regulating the inflammation related to the macrophage polarization in the recovery after SCI.

Milk fat globule-epidermal growth factor-factor 8 (MFG-E8) is a secreted multifunctional glycoprotein [22]. During multiple pathological processes in the CNS, MFG-E8 is upregulated in microglia/macrophages, while astrocytes and neurons can express small amounts of MFG-E8 [23, 24]. Recently, MFG-E8 has been found to play a beneficial role in treating neurodegenerative diseases and traumatic brain injury, inhibit the release of pro-inflammatory mediators, and modulate immune response [25, 26]. Furthermore, MFG-E8 promotes the transition of the microglia phenotype toward the M2 phenotype [27, 28]. Thence, on the basis of the results of proteomic sequencing, we speculate that MFG-E8 may be involved when SCDEs play a part in in inflammatory regulation and macrophage polarization.

This study systematically analyses whether SCDEs can exert an anti-inflammatory effect, which is achieved by inhibiting M1 polarization and promoting M2 polarization of macrophage/microglia, thus promoting the functional recovery after SCI. Meanwhile, MFG-E8 has been verified to be the key component of SCDEs in inflammatory regulation. The knockout (KO) of MFG-E8 in SCs can reverse the anti-inflammatory effects and M2 polarization of macrophage/microglia caused by SCDEs treatment. The SOCS3/STAT3 signaling pathway has been identified to participate in upregulating the M2 polarization induced by MFG-E8. These findings can enrich the mechanism of SCDEs in repairing SCI and provide potential applications and new insights for the clinical translation of SCDE treatment for SCI.

## Results

### Characterizations and uptake of SCDEs

**Fig. 1:**
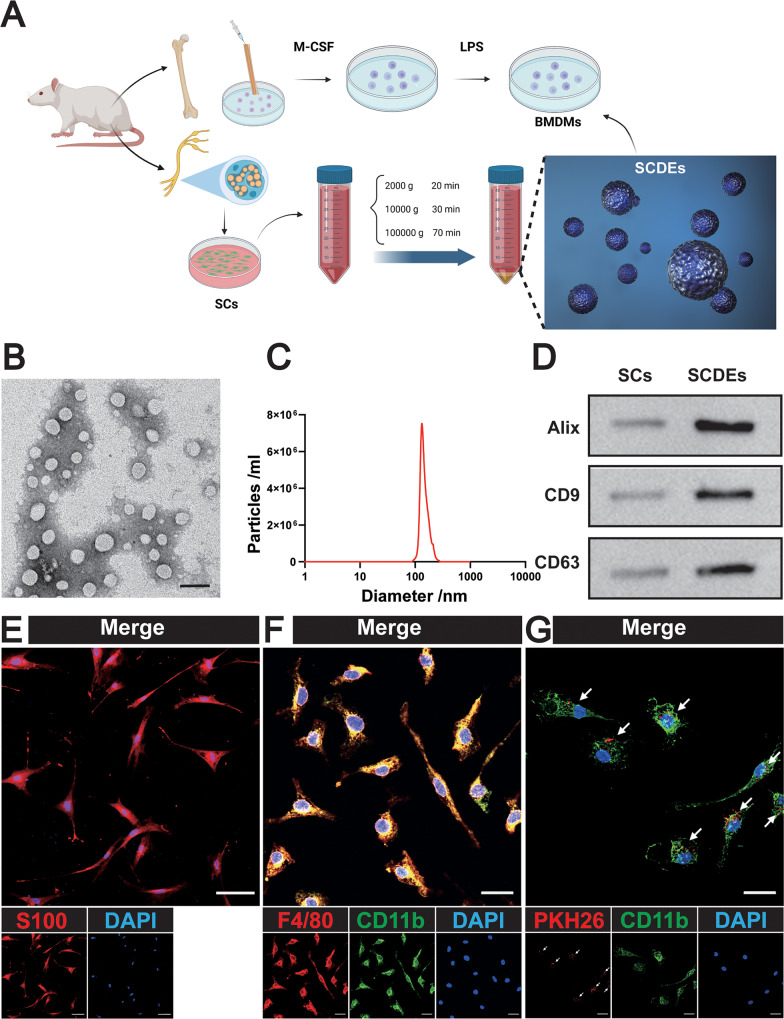
Identification and uptake of SCDEs. **A** Schematic diagram of BMDMs and SCDEs. **B** Morphology of SCDEs under TEM. Scale bars: 100 nm. **C** The diameter distribution of SCDEs. **D** Western blot analysis of the positive expression of Alix, CD9, and CD63 in SCDEs. **E** Representative images of S100 (red). Nuclei were labeled with DAPI (blue). Scale bars: 20 µm. **F** Representative images of F4/80 (red) and CD11b (green). Nuclei were labeled with DAPI (blue). Scale bars: 20 µm. **G** Representative images of PKH26 (red)-labeled SCDEs absorbed by BMDMs. Nuclei were labeled with DAPI (blue). Scale bars: 20 µm.

Primary BMDMs and SCs were extracted from adult female Wistar rats (Fig. 1A). TEM (Transmission Electron Microscopy) was used to observe the morphology of SCDEs, and the results showed that most displayed round or oval shape, with an average diameter of 100 nm (Fig. 1B, C). Three kinds of marker proteins contained in exosomes were detected by western blot: Alix, CD9, and CD63 (Fig. 1D). To identify Schwann cells, immunofluorescence staining was performed on S100 proteins (Fig. 1E). To identify BMDMs, they were stained with F4/80 and CD11b (Fig. 1F). To detect the phagocytosis of BMDM exosomes in vitro, PKH26-labeled SCDEs were co-cultured with BMDMs and the phagocytosis of exosomes was observed via a confocal microscopy (Fig. 1G).

### In vitro SCDEs promoted M2 polarization in LPS-stimulated BMDMs

**Fig. 2:**
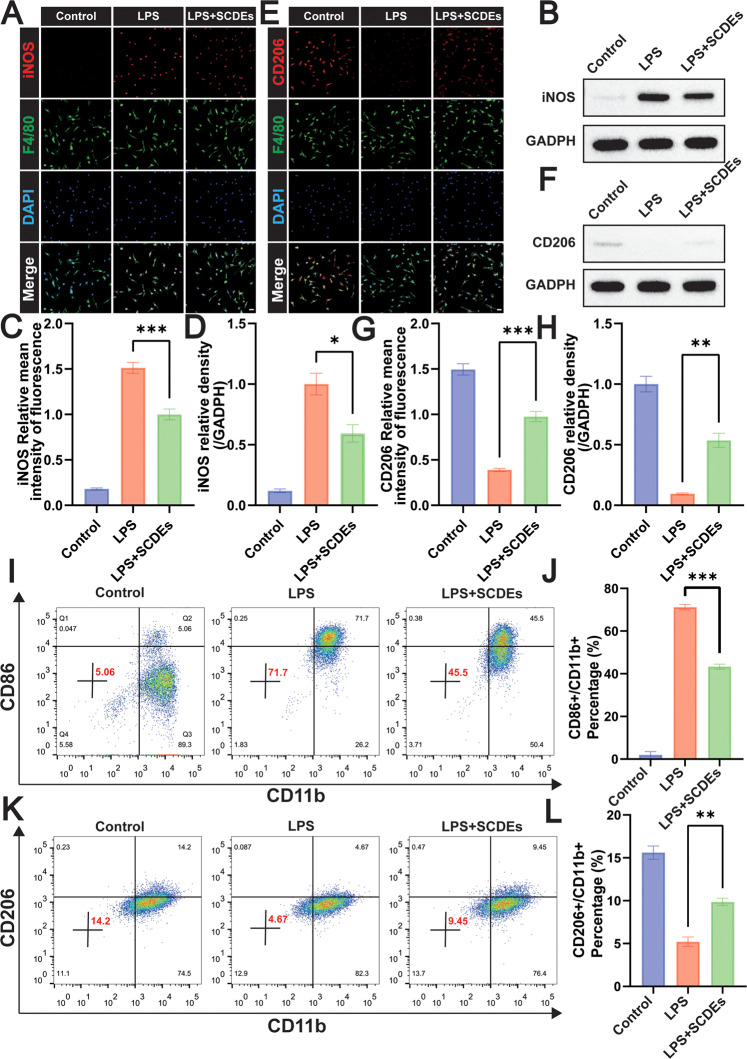
The regulation of SCDEs on M1/M2 polarization in LPS-stimulated BMDMs. **A** Representative images of iNOS (red) and F4/80 (green). Nuclei were labeled with DAPI (blue). Scale bar: 20 μm. **B** Representative western blots showing the reduction of iNOS. **C** The image analysis results were presented as the relative mean intensity of the fluorescence of iNOS (*n* = 5). **D** Quantitative analysis of the iNOS/GAPDH ratio (*n* = 3). **E** Representative images of CD206 (red) and F4/80 (green). Nuclei were labeled with DAPI (blue) in each group (*n* = 5). Scale bar: 20 μm. **F** Representative western blots showing the increase of CD206. **G** The image analysis results were presented as the relative mean intensity of the fluorescence of CD206 (*n* = 5). **H** Quantitative analysis of the CD206/GAPDH ratio (*n* = 3). **I**–**J** Flow cytometry assay detected CD86 + M1 phenotype, indicating that SCDEs treatment suppressed the M1 polarization in LPS-stimulated BMDMs (*n* = 3). **K**, **L** Flow cytometry assay detected CD206 + M2 phenotype, showing that SCDEs treatment promoted the M2 polarization in LPS-stimulated BMDMs (*n* = 3). Data were presented as mean ± SEM. Results were analyzed by One-way ANOVA. Significance: *P < 0.05, **P < 0.01, ***P < 0.001.

A LPS-induced BMDMs inflammation in vitro model was established to further verify the regulation of SCDEs on M1/M2 phenotypes. Immunofluorescence staining was performed on the macrophage marker of F4/80 and the M1 phenotype marker of iNOS (Fig. 2A). The result showed the relative mean intensity of iNOS was significantly reduced in the LPS + SCDEs group (Fig. 2C). Meanwhile, it was confirmed by western blot with the significantly low expression level of iNOS in the LPS + SCDEs group (Fig. 2B, D). Immunofluorescence staining was performed on the macrophage marker of F4/80 and the M2 phenotype marker of CD206 (Fig. 2E), showing that the relative mean intensity of CD206 was obviously enhanced in the LPS + SCDEs group (Fig. 2G). Meanwhile, it was confirmed by western blot with the significantly high expression level of CD206 in the LPS + SCDEs group (Fig. 2F, H). Similarly, flow cytometry detected the M1 phenotype of CD86+ (Fig. 2I, J) and the M2 phenotype of CD206+ (Fig. 2K, L), indicating that SCDE treatment suppressed M1 polarization and promoted M2 polarization in LPS-stimulated BMDMs.

### In vivo SCDEs improved functional recovery after SCI

**Fig. 3:**
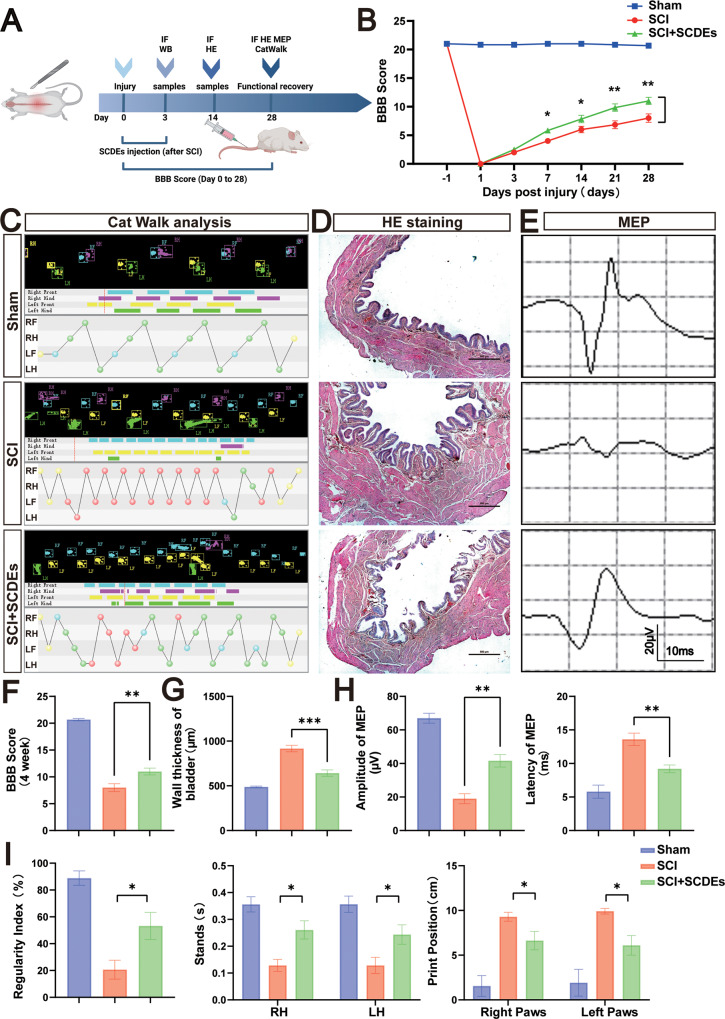
Function recovery of rats by SCDEs after SCI in vivo. **A** The Flow chart of animal experiments. **B**, **F** The BBB scores of Sham, SCI group, and SCI rat group treated with SCDEs (*n* = 5). **C** Representative paw step images and limbs’ supporting timing view of CatWalk gait analysis. **D** The H&E staining of the bladder showed a lesser thickness of the bladder in the SCI rat treated with SCDEs. Scale bar: 500 μm. **E**, **H** Analysis of motor evoked potential (MEP) was performed as an electrophysiological assessment in both groups at day 28 post-injury in both groups (*n* = 5). **G** The wall thickness of the bladder in each group, showing that the bladder function recovered faster in the SCDEs treatment group (*n* = 5). **I** Quantitative analysis of catwalk at day 28 post-injury, including regularity index, print position, and stances of the hindlimb (*n* = 5, RH: right hindlimb, LH: left hindlimb). Data were presented as mean ± SEM. Results were analyzed by One-way ANOVA. Significance: *P < 0.05, **P < 0.01, ***P < 0.001.

To assess the functional recovery after SCI, the BBB score was observed by two trained observers at 1 day before, and 1, 3, 7, 14, 21, and 28 days after SCI. The BBB score of the SCI + SCDEs group was significantly higher (Fig. 3B, F). The results of gait analysis showed that the rear limb coordination of the SCI + SCDEs group was greatly improved, especially in the regularity index, print position, and stance of the hindlimb (Fig. 3C, I). Meanwhile, the H&E staining of the bladder showed a lesser thickness of the bladder in the SCI rat treated with SCDEs, which meant that the functions of the bladder recovered faster in the SCDEs treatment group (Fig. 3D, G). To assess the improvement in nerve conduction, electrophysiological assay was performed on rats. The MEP results showed that the MEP latency was shortened and the amplitude increased in the SCI + SCDEs group (Fig. 3E, H). These results suggested that SCDEs promoted the functional recovery after SCI.

### The role of SCDEs in improving spinal cord repair after SCI

**Fig. 4:**
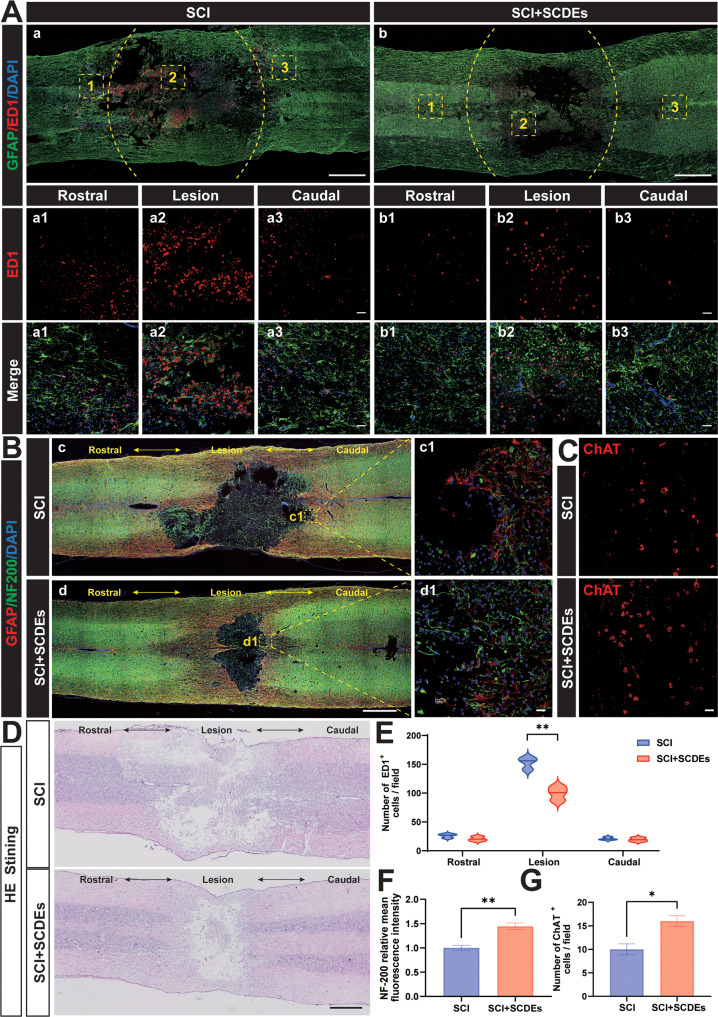
SCDEs improved spinal cord repair after SCI in vivo. **A** Representative images of immunofluorescence staining of ED1 (red) and GFAP (green) in each group at day 3 after SCI (*n* = 3). Scale bars: 1000 µm. The bottom row of images were high-resolution versions of the boxed regions in the top row of images. Scale bars: 50 µm. **B** Representative images of immunofluorescence staining of GFAP (red) and NF-200(green) in each group at day 14 after SCI (*n* = 3). Scale bars: 1000 µm. The right images were high-resolution versions of the boxed regions in the left images. Scale bars: 20 µm. **C** Representative images of immunofluorescence staining of ChAT(red) in the SCI group and SCI + SCDEs group (*n* = 3). Scale bars: 20 µm. **D** The H&E staining of the spinal cord showed the reduction of the damaged area in the SCI rat treated with SCDEs. **E** Quantitative analysis of the number of ED1 + cells, showing that being treated with SCDEs can reduce the number of ED1 + cells in the rostral, lesion, and caudal. **F** Quantitative analysis of the relative mean intensity of the fluorescence of NF200. **G** Quantitative analysis of the number of ChAT+ cells, showing that the number of ChAT+ cells in the SCI rat treated with SCDEs was higher. Data were presented as mean ± SEM. Results were analyzed by Student’s *t*-test. Significance: *P < 0.05, **P < 0.01.

In order to preliminarily explore the mechanism of SCDEs repairing spinal cord injury, immunofluorescence staining of ED1 (a specific marker of activated macrophages/microglia) was performed. The results showed that the macrophages/microglia infiltration was reduced in the SCI + SCDEs group (Fig. 4A, E). Immunofluorescence staining of GFAP (red) and NF-200 (green) in each group on day 14 after SCI was performed to evaluate the injured area of the spinal cord and nerve survival levels (Fig. 4B), which showed that after SCDEs treatment, the recovery of lesion volume and the relative mean intensity of NF-200 were better than those of the SCI group (Fig. 4F). Choline acetyltransferase (ChAT) is the most specific indicator for monitoring the functional status of cholinergic neurons in the CNS. SCDEs significantly improved motor function after SCI, so immunofluorescence staining of ChAT was performed (Fig. 4C) and the results showed that the ChAT-positive cells significantly increased in number after the treatment of SCDEs (Fig. 4G). The H&E staining of the spinal cord showed that the damaged area in the SCI rat was reduced after being treated with SCDEs (Fig. 4D).

### SCDEs modulated macrophage/microglia polarization towards M2 phenotype in vivo

**Fig. 5:**
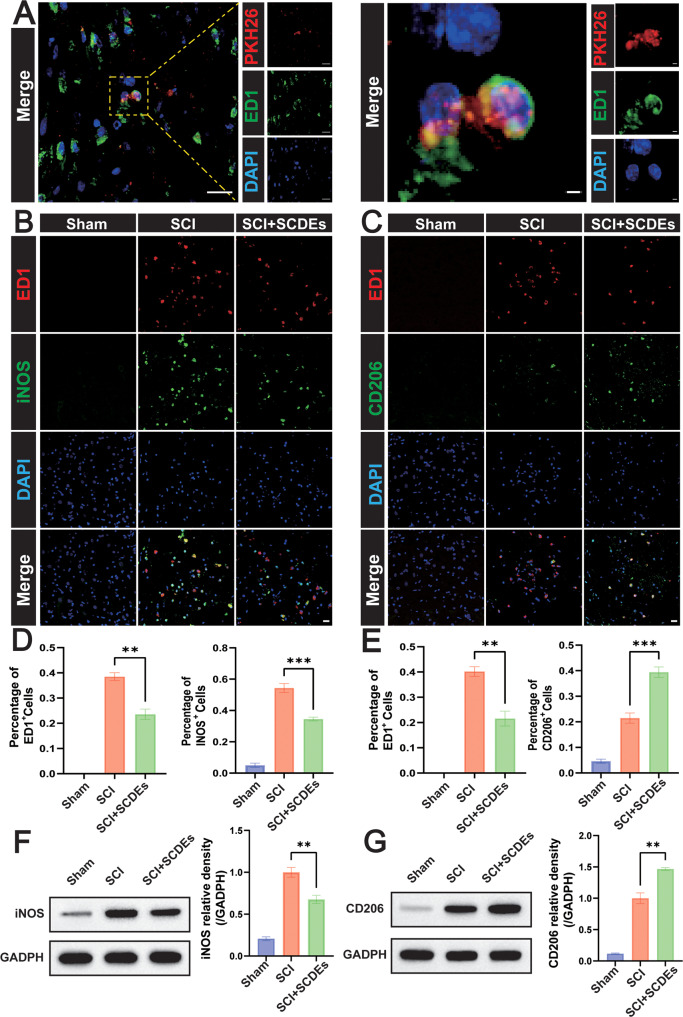
The effect of SCDEs on the regulation of M1/M2 polarization in vivo. **A** Representative images of PKH26 (red)-labeled SCDEs absorbed by macrophages/microglia in vivo. Scale bars: 20 µm. The right images were high-resolution versions of the boxed regions in the left images. Scale bars: 2 µm. **B** Representative immunofluorescence staining images of iNOS (green) and ED1 (red). Nuclei were labeled with DAPI (blue) in each group (*n* = 3). Scale bar: 20 μm. **D** Quantitative analysis of the ED1+ cells and iNOS+ cells (*n* = 3). **F** Representative western blots showing the reduction of iNOS in vivo (*n* = 3). **C** Representative immunofluorescence staining images of CD206 (green) and ED1 (red). Nuclei were labeled with DAPI (blue) in each group (*n* = 3). Scale bar: 20 μm. **E** Quantitative analysis of the ED1-positive cells and CD206-positive cells (*n* = 3). **G** Representative western blots showing the increase of CD206 in vivo (*n* = 3). Data were presented as mean ± SEM. Results were analyzed by One-way ANOVA. Significance: *P < 0.05, **P < 0.01, ***P < 0.001.

PKH26-labeled SCDEs were injected via the tail vein and were uptaken by macrophage/microglial cells in vivo (Fig. 5A). SCDEs can reduce the number of infiltrated macrophages/microglia in vivo, but the polarization direction was not clear. To clarify the M1 polarization of macrophages/microglia, immunofluorescence staining was performed on the macrophage/microglia marker of ED1 and the M1 phenotype marker of iNOS (Fig. 5B), which showed that the positive cells of ED1 and iNOS were significantly reduced (Fig. 5D). Also, as verified by western blot, the expression level of iNOS in the SCI + SCDEs group was significantly lower (Fig. 5F). On the other hand, to clarify the M2 polarization of macrophages/microglia, immunofluorescence staining was performed on the macrophage/microglia marker of ED1 and the M2 phenotype marker of CD206 (Fig. 5C), which indicated that the positive cells of ED1 and CD206 were significantly reduced in the SCI + SCDEs group and that the proportion of CD206+ cells was obviously increased (Fig. 5E). Also, as verified by western blot, the expression level of CD206 in the SCI + SCDEs group was significantly higher (Fig. 5G).

### MFG-E8 Was the Key Component of SCDEs in Regulating Inflammatory Responses

**Fig. 6:**
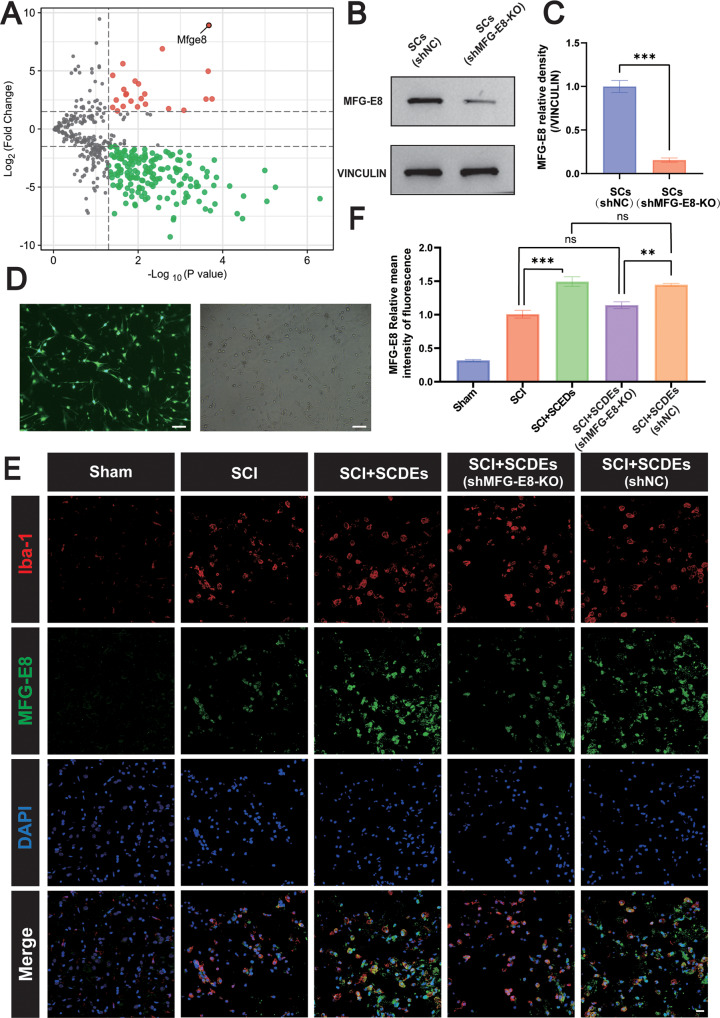
MFG-E8 was the key component of SCDEs in the regulation of inflammatory responses. **A** The differentially expressed proteins between SCDEs and SCs were analyzed by proteomics, and the up-regulated or down-regulated proteins of SCDEs were screened and compared with SCs. **B** Representative western blots showing the knockout of MFG-E8 in the SCs (shNC) and SCs (shMFG-E8-KO) group. **C** Quantitative analysis of the MFG-E8/VINCULIN ratio in the SCs (shNC) and SCs (shMFG-E8-KO) group (*n* = 3). **D** Representative fluorescence images of SCs (green) after the MFG-E8 gene was knocked out. Scale bar: 100 μm. **E** Representative immunofluorescence staining images of Iba-1 (red) and MFG-E8 (green). Nuclei were labeled with DAPI (blue) in each group (*n* = 5). Scale bar: 20 μm. **F** The image analysis results were presented as the relative mean intensity of the fluorescence of MFG-E8. Data were presented as mean ± SEM. Results were analyzed by Student’s t-test and One-way ANOVA. Significance: **P < 0.01, ***P < 0.001.

Exosomes contain different biologically active molecules, including RNAs, proteins, and lipids. In order to explore the key components in exosomes related to regulating the inflammatory responses, we performed differential proteomic analysis between SCDEs and SCs and screened out MFG-E8 as a protein that was highly expressed in SCDEs compared with SCs (Fig. 6A). To verify whether MFG-E8 plays a key role in regulating inflammation, we knocked out the MFG-E8 in SCs via lentiviral transfection, which was proved successful by GFP fluorescence (green) (Fig. 6D). Next, we confirmed the successful knockout of MFG-E8 in SCs via western blot (Fig. 6B, C). In order to further verify whether the content of MFG-E8 protein in SCDEs(MFG-E8-KO) decreased, SCDEs extracted from SCs (MFG-E8-KO) were injected into the rats via the tail vein and immunofluorescence staining was performed to observe the changes of the content of MFG-E8. Finally, the immunofluorescence staining of MFG-E8 showed that MFG-E8 was significantly decreased in the SCDEs (shMFG-E8-KO) (Fig. 6E, F).

### The knockout of MFG-E8 suppressed the M2 polarization in vitro and in vivo

**Fig. 7:**
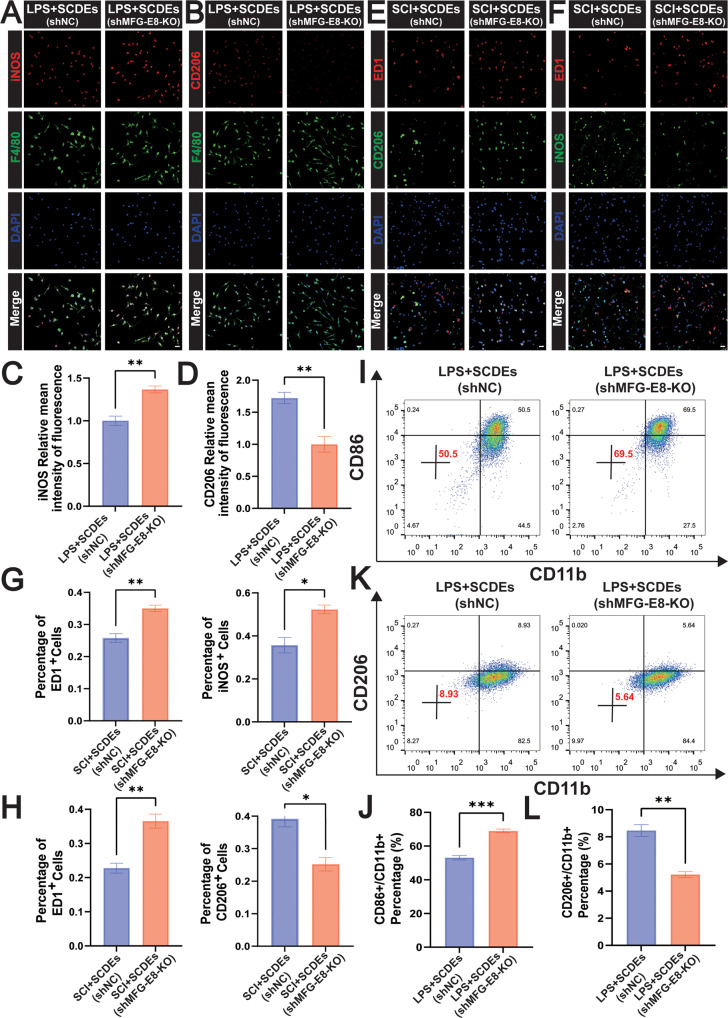
MFG-E8 knockout suppressed the M2 polarization in vitro and vivo. **A** Representative immunofluorescence staining images of iNOS (red) and F4/80 (green) in vitro. Nuclei were labeled with DAPI (blue) in each group (*n* = 5). Scale bar: 20 μm. **B** Representative immunofluorescence staining images of CD206 (red) and F4/80 (green) in vitro. Nuclei were labeled with DAPI (blue) in each group (*n* = 5). Scale bar: 20 μm. **C**, **D** The results of the iNOS and CD206 relative mean intensity of the fluorescence (*n* = 5). **E** Representative immunofluorescence staining images of iNOS (green) and ED1 (red) in vivo. Nuclei were labeled with DAPI (blue) in each group (*n* = 3). Scale bar: 20 μm. **F** Representative immunofluorescence staining images of CD206 (green) and ED1 (red) in vivo. Nuclei were labeled with DAPI (blue) in each group (*n* = 3). Scale bar: 20 μm. **G**, **H** Quantitative analysis of the positive cells of ED1, iNOS, and CD206 (*n* = 3). **I**, **J** Flow cytometry assay detected CD86+/CD11b + M1 phenotype, indicating that MFG-E8 knockout promoted the M1 polarization (*n* = 3). **K**, **L** Flow cytometry assay detected CD206+/CD11b + M2 phenotype, showing that MFG-E8 knockout inhibited the M2 polarization (*n* = 3). Data were presented as mean ± SEM. Results were analyzed by One-way ANOVA. Significance: *P < 0.05, **P < 0.01, ***P < 0.001.

We further analyzed the regulatory effect of MFG-E8 on macrophage/microglia polarization in vitro and in vivo. Concerning the model of LPS-induced inflammation in BMDMs, immunofluorescence staining was performed on the macrophage marker of F4/80, the M1 phenotype marker of iNOS, and the M2 phenotype marker of CD206 (Fig. 7A, B). The result showed that the relative mean intensity of the fluorescence of iNOS was significantly higher and the relative mean intensity of the fluorescence of CD206 was significantly lower in the LPS + SCDEs (shMFG-E8-KO) group (Fig. 7C, D). That is to say, the knockout of MFG-E8 attenuated the M2 Polarization of BMDMs in vitro. Similarly, immunofluorescence staining was performed on the macrophage marker of ED1, the M1 phenotype marker of iNOS, and the M2 phenotype marker of CD206 in vivo. (Fig. 7E, F). The result showed that the iNOS+ cells were higher and the CD206+ cells were lower in the SCI + SCDEs (shMFG-E8-KO) group (Fig. 7G, H). That is to say, the knockout of MFG-E8 attenuated the M2 Polarization in vivo. Also, flow cytometry detected CD86 + M1 phenotype, indicating that MFG-E8 knockout promoted M1 polarization (Fig. 7I, J) while inhibiting M2 polarization (Fig. 7K, L).

### Improved Inflammatory Microenvironment Inhibited Neuronal Apoptosis in Vitro

**Fig. 8:**
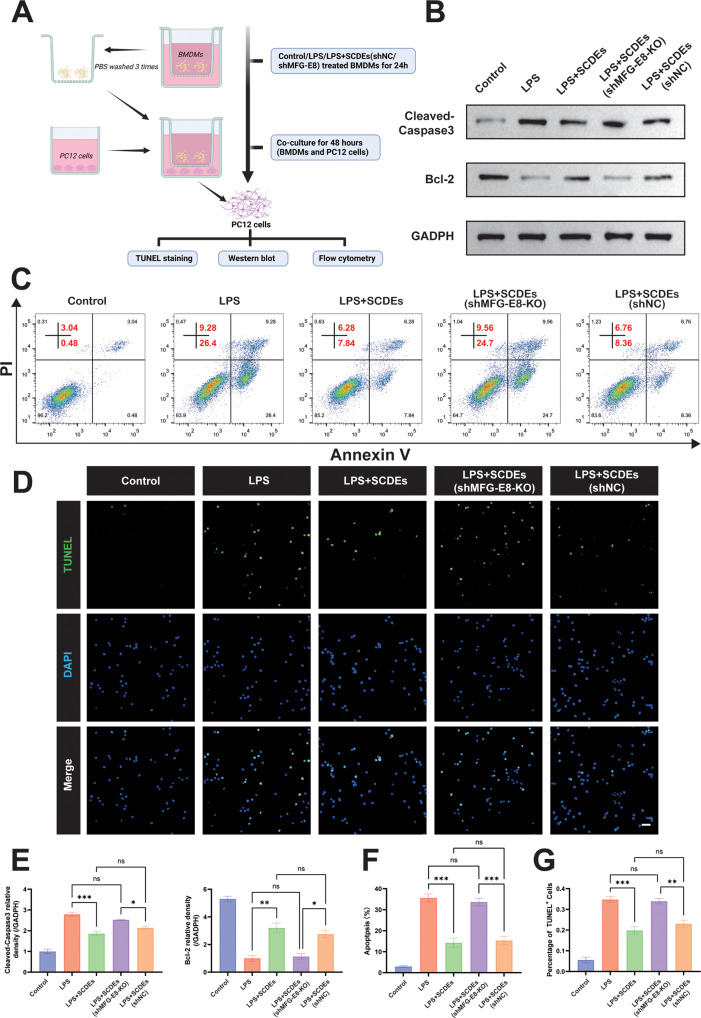
Improved inflammatory microenvironment inhibited neuronal apoptosis. **A** Schematic representation of co-culture of PC12 cells and BMDMs. **B**, **E** Representative western blots of Cleaved Caspase-3 and Bcl-2 in PC12 cells. Quantitative analysis of the Cleaved Caspase-3/GAPDH ratio and Bcl-2/GAPDH ratio in PC12 cells (*n* = 3). **C**, **F** Flow cytometry assay detected apoptosis of PC12 cells in each group, showing that SCDEs inhibited LPS-induced apoptosis and that the knockout of MFG-E8 reversed the positive effect (*n* = 3). **D**, **G** The TUNEL staining of PC12 cells showed that SCDEs reduced TUNEL + cells while MFG-E8 knockout increased TUNEL + cells (*n* = 3). Scale bar: 50 μm. Data were presented as mean ± SEM. Results were analyzed by One-way ANOVA. Significance: ns-not significant, *P < 0.05, **P < 0.01, ***P < 0.001.

To explore whether SCDEs affect neuronal apoptosis after the inflammatory microenvironment is improved, PC12 cells were co-cultured with BMDMs (Fig. 8A). After being co-cultured for 24 h, PC12 cells were isolated for apoptosis assay. The results of the western blot showed that SCDEs reduced the expression of Cleaved Caspase 3 and increased the expression of Bcl-2 in PC12 cells, and that the knockout of MFG-E8 reversed the anti-apoptosis effect (Fig. 8B, E). Similarly, flow cytometry also demonstrated the same result (Fig. 8C, F). The TUNEL staining showed that SCDEs reduced TUNEL + cells, while MFG-E8 knockout increased TUNEL + cells in PC12 cells (Fig. 8D, G).

### M2 Polarization Induced by MFG-E8 Knockout in SCs Was Upregulated via SOCS3/STAT3 Signaling Pathway

**Fig. 9:**
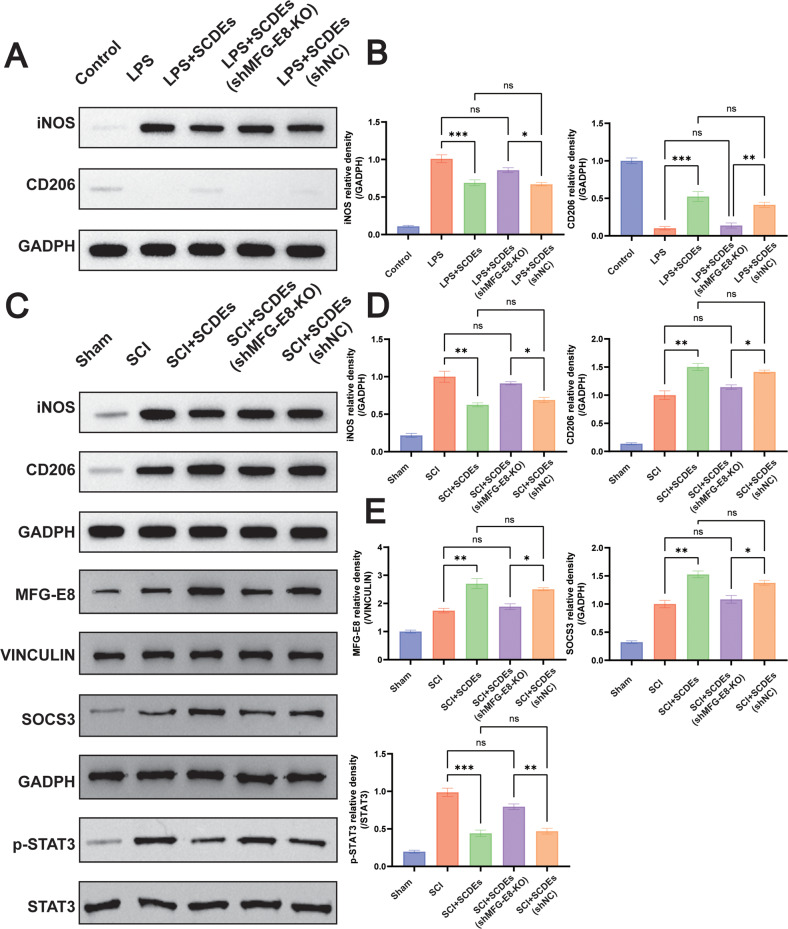
M2 polarization induced by MFG-E8 in SCDEs was upregulated via SOCS3/STAT3 signaling pathway. **A** Representative western blots showed iNOS was reduced, and CD206 was increased, while MFG-E8 knockout reversed the effects of SCDEs in vitro. **B** Quantitative analysis of the iNOS/GAPDH and CD206/GAPDH ratio (*n* = 3). **C** Representative western blots showing the reduction of iNOS and the increase of CD206, while MFG-E8 knockout reversed the effects of SCDEs in vivo. Representative western blots showed that SOCS3/STAT3 signaling was activated via MFG-E8 in SCDEs, while MFG-E8 knockout reduced the SOCS3 and increased the STAT3 in vivo. **D** Quantitative analysis of the iNOS/GAPDH and CD206/GAPDH ratio in vivo (*n* = 3). **E** Quantitative analysis of the MFG-E8/GAPDH, SOCS3/GAPDH, and STAT3/GAPDH ratio and CD206/GAPDH ratio in vivo (*n* = 3). Data were presented as mean ± SEM. Results were analyzed by One-way ANOVA. Significance: ns-not significant, *P < 0.05, **P < 0.01, ***P < 0.001.

In order to find out the detailed molecular mechanism of MFG-E8 in regulating the M2 polarization of macrophages/microglia, western blot was performed. It was found that the SOCS3/STAT3 signaling pathway played an important part in upregulating M2 polarization induced by MFG-E8 knockdown (Fig. 9). The MFG-E8 in SCDEs reduced the expression of iNOS and increased the expression of CD206, indicating that MFG-E8 suppressed M1 polarization and promoted M2 polarization, while MFG-E8 knockout reversed the effects of SCDEs in vitro (Fig. 9A, B). Similar results were verified in vivo (Fig. 9C, D). Meanwhile, it was found that MFG-E8 could reduce the expression of phosphorylated STAT3 when the expression of SOCS3 was increased, while MFG-E8 knockout could reduce SOCS3 and increase the phosphorylated STAT3 in vivo (Fig. 9C, E).

## Discussion

**Fig. 10:**
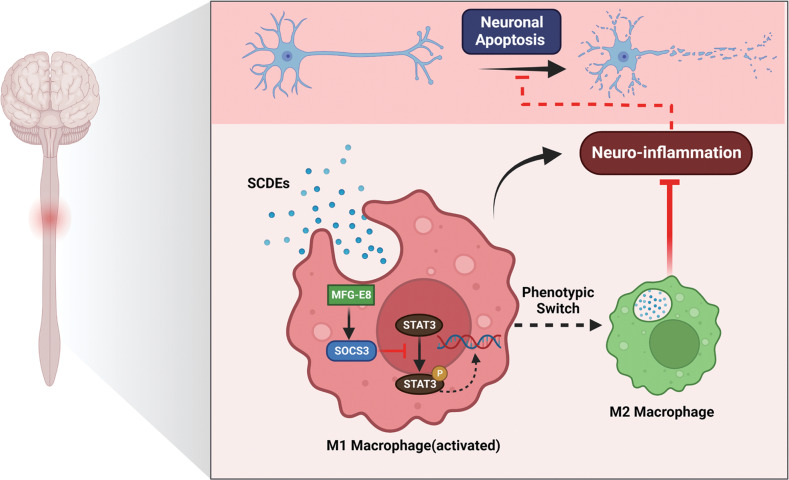
The mechanism of SCDEs containing MFG-E8 modify macrophage/microglial polarization through SOCS3/STAT3 pathway after SCI. Spinal cord injury induced activation of STAT3 phosphorylation in macrophages/microglia, led to M1 polarization and exacerbated inflammatory damage at the site of spinal cord injury. After treatment with SCDEs, they were phagocytosed by macrophages/microglia at the injury site and began to release MFG-E8. This increased the protein levels of SOCS3, which inhibited the phosphorylation of STAT3 to promote the conversion of the macrophage/microglia phenotype to M2 for alleviating inflammatory damage. Moreover, the improved inflammatory microenvironment at the SCI site reduced neuronal apoptosis and promoted nerve regeneration.

SCI is a devastating disease that can cause severe dysfunction of motor, sensory, and autonomic nerves, thus imposing a huge economic burden and exerting a great affect on the quality of life [1, 2]. Therapies-influenced inflammatory cascades are likely to benefit the functional recovery after SCI. Our study demonstrates that SCDEs can attenuate the inflammatory response by regulating macrophage/microglia polarization, reducing neuronal apoptosis, and promoting the functional recovery after SCI. Moreover, MFG-E8 is the key component of SCDEs in improving inflammation. KO of MFG-E8 can partly reverse the anti-inflammatory effects and promote M2 polarization of macrophage/microglia via the SOCS3/STAT3 signaling pathway (Fig. 10). These results can enrich the anti-inflammatory mechanism of SCDEs treatment in repairing SCI and provide a new potential and therapeutic option for repairing SCI.

Exosomes have been verified to promote neuronal repair and growth and modulate inflammation responses in many studies [29]. We have recently published the finding that SCDEs can promote the functional recovery after SCI by increasing autophagy and decreasing apoptosis, inducing axonal protection, decreasing the deposition of chondroitin sulfate proteoglycan (CSPG) and their detailed molecular mechanism. That is to say, SCDEs transplantation has a potential application prospect in the treatment of SCI [20, 21]. In our study, it was evident that the histological levels in rats after spinal cord injury were recovered after tail vein injection of SCDEs, especially the bladder tissue function. After SCI, long-term dysuria and urine retention will lead to the hypertrophy of the bladder wall with a high ratio of collagen to muscle [30, 31]. It was found that the thickness of the bladder wall after SCI was correlated with the recovery of bladder function, and that the mechanism might cause changes in altered c-fiber efferent activity [31–33] and the expression of some protein receptors [34–37]. Our electrophysiological assay has shown that SCDEs can promote the recovery of nerve conduction function after SCI by inhibiting inflammation, which may involve an improvement in the activity of C-afferent fibers. In addition, some receptors on the bladder wall or detrusor play an important role in inhibiting bladder wall fibrosis. Some of the key components contained in SCDEs administered through the tail vein are likely to act on some receptors (such as VEGF, FGF, and PDGF) through systemic blood, thus promoting the recovery of bladder function. However, further experiments are still needed to verify the exact mechanism. A growing number of studies have confirmed that exosomes have a positive effect in reducing inflammation [38–40]. However, few studies have been focused on the key protein in SCDEs that is closely related to inflammation inhibition and its specific mechanism for SCI repair. The present study has identified MFG-E8 as the key component of SCDEs in improving the anti-inflammatory effects and promote the M2 polarization of macrophage/microglia caused by SCDEs treatment.

MFG-E8 is a secreted multifunctional glycoprotein. It can be expressed in various cells and play an important role in treating different diseases [41]. An increasing amount of research has found that MFG-E8 plays a neuroprotective role by inhibiting neuronal apoptosis and reversing anti-inflammatory effects in various neurodegenerative diseases and traumatic brain injury [42]. MFG-E8 can inhibit the release of pro-inflammatory mediators to regulate immune responses and promote the reprogramming of microglial, and convert the microglia phenotype to the M2 phenotype [25–28]. Elodie Segura et al. have revealed that mature dendritic cells can also secrete exosomes containing MFG-E8 by conducting proteomic and biochemical analyses [43]. However, it has not been studied that Schwann cell-derived exosomal MFG-E8 across the BBB is applied for SCI [24]. Herein, it has been verified that MFG-E8 is the key component of SCDEs in improving the inflammatory response by proteomic sequencing. Besides, SCDEs treatment with MFG-E8 knockout has attenuated the anti-inflammatory effect with a higher number of ED1-positive cells and suppressed the M2 polarization with a lower number of CD206-positive cells. Similarly, Shi et al. have found that MFG-E8 could regulate the alteration of microglia M1/M2 phenotypes in AD [27]. Wu et al. Have found that M2 phenotype marker is reduced and M1 phenotype marker is increased after MFG-E8 is knocked out in microglia [28]. Neuronal apoptosis is another important molecular pathological feature in SCI [2]. The interactions between microenvironmental inflammation responses and neuronal apoptosis are complex [44]. Many studies have shown that exosomes can also play a role in inhibiting apoptosis [45]. Our study has proved that SCDEs can inhibit apoptosis and the knockout of MFG-E8 can reverse the positive effect in vivo and in vitro, further explaining the mechanism of SCDEs in repairing SCI from another perspective.

Macrophage polarization in CNS has emerged during the past decades and is largely generated by the local microenvironmental signals [46]. It means that macrophages are able to switch from pro-inflammatory M1 phenotype to anti-inflammatory M2 phenotype [5, 6]. M2 macrophages are considered to be critical in attenuating the inflammation response and promoting tissue remodeling. The targeted interventions have a great application prospect for the treatment of SCI [9], such as blocking the recruitment and proliferation of macrophages, inhibiting the activation pathway of M1 phenotype, and promoting the transformation to M2 macrophages. A previous study has found that increased expression of MFG-E8 can downregulate the phosphorylation of STAT3 and upregulate the expression of SOCS3, then promote M2 polarization and exert an anti-inflammatory effect after subarachnoid hemorrhage [47]. Nevertheless, it is still unclear whether the mechanism of MFG-E8 in SCDEs mediates the shift of macrophage/microglia to M2 after SCI. In our study, the KO of MFG-E8 in SCDEs may induce the activation of SOCS3 and interfere with the phosphorylation of STAT3 to stimulate M2 polarization and reduce inflammation.

There are several limitations to our study. This research is mainly focused on the inflammation modulation of SCDEs for SCI treatment without analyzing the neural proliferation and differentiation and axonal regeneration. In addition, the study has only confirmed that MFG-E8 can induce M2 polarization without exploring the multiple activated M2 subtypes, including M2a, M2b, and M2c. Further studies involved in knockout rats/mice are required to verify the above findings, since only shRNAs were used to knock out MFG-E8 in our study.

In conclusion, SCDEs can exert an anti-inflammatory effect by inhibiting M1 polarization and promoting M2 polarization of macrophage/microglia, and reduce neuronal apoptosis and improve the recovery of motor function after SCI, which may be ascribed to the MFG-E8 in SCDEs mediated by regulating SOCS3/STAT3 signaling pathway. These findings can enrich the anti-inflammatory mechanism of SCDEs in repairing SCI and provide potential application prospects and new insights for the clinical translation of SCDEs for SCI.

## Materials and methods

### Preparation of animals and experimental groups

All adult female Wistar rats (the body weight ranging from 200 to 220 g, *n* = 200) were supplied by Beijing Vital River Laboratory Animal Technology Co., Ltd. (Beijing, China, Permission Number: SCXK (Jing)-2016-0011). All female Wistar rats were housed in the same environments (the temperature ranging from 22 to 24 °C, the humidity ranging from 60 to 80%). All animal experimental protocols were approved by the Ethics Committee of the Institute of Radiation Medicine, Chinese Academy of Medical Sciences (Tianjin, China, Approval Number: IRM-DWLL-2021180). Rats were randomly divided into the following groups: Sham, SCI, SCI + SCDEs, SCI + SCDEs (shMFG-E8-KO), and SCI + SCDEs (shNC). The treatment groups were injected with 250 uL of SCDEs (0.1 ug/uL) transfected with lentivirus, and the SCI group was injected with the same volume of DPBS. Half an hour after the spinal cord injury model was established, SCDEs were injected through the tail vein of rats. We injected rats used in the acute phase (3 days) with SCDEs once a day. Rats in the subacute (2 weeks) and postacute phases (4 weeks) were injected with SCDEs via the tail vein three times per week.

### Cell culture

Bone marrow was extracted from the femur and tibia of a female Wistar rat. It was incubated in the induction medium containing DMEM/F-12 (Gibco, # 11320033), FBS (10%, Gibco, #10099141 C), penicillin/streptomycin (1%, Gibco, #15070063), and M-CSF (20 ng/ml, #SRP3247, Sigma-Aldrich) at 37 °C in a 5% CO_2_ incubator for 7 days. After 7 days of induction, BMDMs could be defined as M0 macrophages and set as the control group, which could be used for subsequent experiments. M0 macrophages were incubated under the condition of LPS (1000 ng/ml, #L3129, Sigma-Aldrich) for 24 h and set as the LPS group. M0 macrophages were incubated under the conditions of LPS and SCDEs (2 µg/ml) for 24 h and set as the SCI + SCDEs group.

Primary SCs were extracted from the sciatic nerve of rats. The sciatic nerve was isolated and minced, digested with 0.3% of type II collagenase (Solarbio, #C8150, Beijing, China) and TrypLE (Gibco, #2277057, Denmark). The cells were cultured in SC purification medium (10% of FBS, 10 μM of arabinoside hydrochloride (Ara-C, Solarbio, #C1768, Beijing, China), 1% of penicillin/streptomycin, DMEM/F-12) at 37 °C in a 5% CO_2_ incubator for 24 h to remove fibroblasts. Then SC growth medium was used at 37 °C in a 5% CO_2_ incubator to continue the culture.

PC12 cells were obtained from Shanghai Institutes for Biological Science, Chinese Academy of Sciences. The cells were grown in a medium containing DMEM (Gibco, # 12430054), FBS (10%), and penicillin/streptomycin (1%) at 37 °C in a 5% CO_2_ incubator cultured under conditions. To study the effect of BMDMs polarization on neuronal apoptosis, we co-cultured BMDMs with PC12 cells in a 0.4 μm-pore Transwell^®^ coculture system (Corning, USA). BMDMs were seeded in the upper chamber (1 × 10^5 per chamber), which were then treated with PBS, LPS, LPS + SCDEs, LPS + SCDEs (shNC), or LPS + SCDEs (shMFG-E8-KO) for 24 hours. The inserts were gently washed three times with PBS and placed above the PC12 cells (1 × 10^5 per well) in 24-well plates. Finally, PC12 cells were collected after 48 hours for apoptosis analysis.

### The preparation, purification, and identification of SCDEs

When Schwann cells were cultured to the P3-P5, and exosome-free serum was used to prepare the Schwann cell medium. During the cell culture process, the medium was changed every three days. The medium was collected into the centrifuge tube and centrifuged at 4 °C and 300 × *g* for 5 min to remove dead cells from the medium. Then, the supernatant of the medium was placed at −20 °C for use. After the Schwann cell culture medium was collected, Schwann cell exosomes were obtained through this experiment through ultracentrifugation. In the first step, centrifugation was performed at 4 °C and 2000 × *g* for 20 min to remove cell residues and organelles, and the supernatant was collected. In the second step, centrifugation was performed at 4 °C and 10000 × *g* (Centrifuge 5810R, Eppendorf, Germany) for 60 min. Subsequently, the collected supernatant was filtered with a 0.22-μm cell filter, and the supernatant was collected for the third time, centrifuged at 130,000 × g and at 4 °C (Beckman Optimal-100 XP, Beckman Coulter, Germany) for 70 min. After the final ultracentrifugation, SCDEs were resuspended in sterile water for the following experiments. The total protein concentration of exosomes was quantified by BCA assay (Thermo Scientific, #23225 MA, USA). After a series of sample preparation processes, the morphology of exosomes was observed by using a high-resolution transmission electron microscope (TEM, Hitachi HT7700, Tokyo, Japan). The SCDEs suspension was diluted 1000-fold, and the samples were examined by NanoSight (Malvern, UK, NS300) at 25 °C. Western blot was performed to identify specific exosome surface protein markers, such as Alix CD9 and CD63.

### SCDEs uptaken by BMDMs

According to the instructions of the reagent instructions of the PKH26 Red Fluorescent Cell Linker Mini Kit (MINI26-1KT, #MKCM1863, Sigma-Aldrich), SCDEs were incubated with excess dye at room temperature for 4 h and then removed by ultracentrifugation at 100,000 × *g* for 1 h. After being co-cultured with PKH26-labeled exosomes for 16 h, BMDMs were fixed, penetrated, blocked, and incubated with CD11b antibody (1:400; Abcam, #1211 MA, USA) overnight at 4 °C, and then incubated with secondary antibody. After being washed, the slides were removed from the 24-well plate and mounted with Mounting Medium with DAPI (Abcam, #104139, MA, USA). PKH26-labeled SCDEs being uptaken by primary bone marrow-derived macrophages was observed by the ultra-high resolution laser confocal microscope (ZEISS LSM 900, Germany).

### Lentivirus production and cell transfection

LV-MFGE8-EGFP inhibitor vector was constructed from the lentiviral vector (GeneChem, shanghai, China). Negative control was constructed by LV empty lentivirus (LV-NC-EGFP). The short hairpin RNA (shRNA) sequences of MFG-E8 protein (shRNA-MFG-E8) and negative control (shRNA-NC) were designed and synthesized by GeneChem (Shanghai, China). HitransG P Infection Enhancement Reagent (25×) was used to transfect cells at the multiplicity of infection (MOI) value of 50. After the cultured primary SCs were infected with these lentiviruses, the SCs cell growth medium was replaced. The culture was continued for 72 h to observe the transfection efficiency. Brightfield and fluorescence images were taken with a fluorescence microscope. The knockout efficiency of the target protein was evaluated via western blot.

### Contusion model of spinal cord injury in rats

The modified Allen method was used to construct the contusion model of SCI in rats, and stability and uniformity of the model were well maintained [48]. After being weighed, the rats were anesthetized with 5% of isoflurane (RWD lifescience, Shenzhen, China). A dorsal laminectomy was performed on the T10 vertebral body to expose the spinal cord. The impact bar was placed on the spinal cord, and a 10 g node (the diameter of 2.5 mm) was freely dropped from a height of 2.5 cm to create contusion injury with NYU Impactor Model III (W.M. Keck Center for Collaborative Neuroscience, Rutgers, the State University of New Jersey, United States). After hemostasis, the muscle and skin at the incision site is sutured. Successful SCl exhibits the following characteristics in the rats: formation of tail sway reflex, spinal cord ischemia, leg swings, and paralysis^[49]^. If rats did not show the above symptoms after a single blow and the motor function of their hind limb remained normal on the second day after surgery, they were considered unsuccessful in the construction of a spinal cord injury model and would be excluded from the scope of this study. Within 3 days after SCI, cefuroxime sodium was used to prevent wound infection. Within 7 days after SCI, the bladder was squeezed through artificial assistance to void 3 times a day until the reflexive control of bladder function recovered.

### Hematoxylin and Eosin (H&E) staining

After the paraffin sections were prepared, they were heated in a constant temperature oven at 60 °C for 4 h. Then, the sections were placed in xylene I and II for 10 min and placed in alcohol solutions with different concentrations, including 100%, 95%, 90%, 80%, and 70%, for 5 min each time, and rinsed in distilled water for 1 min. The sections were stained with the hematoxylin (Solarbio, #G1120, Beijing, China) staining solution for 2 min, differentiated with the differentiation solution (1% of hydrochloric acid), washed in distilled water again for 2 min, and then dyed with 0.5% of the eosin staining solution (Solarbio, #G1120, Beijing, China) for 1 min. Finally, in order to complete the dehydration, the tissue sections were successively placed in 70%, 80%, 90%, and 100% gradient ethanol solution. The slides were placed in xylene I and II for 5 min and were sealed with neutral resin (Solarbio, # G8590, Beijing, China). Samples were observed and photographed on a fully automated tissue in situ multi-label landscape quantification analyzer (Vectra Polaris, PerkinElmer).

### Behavioral evaluation

The BBB functional score was evaluated to assess the recovery of motor function. The score was evaluated at the same time before the surgery, on the day after surgery, on day 3, on day 7, and on the same day every week. The test was performed by two trained researchers (blinded to treatment) to observe and assess the motor function of the rats. The Catwalk-assisted gait analysis (Noldus Information Technology B.V, Netherlands) was performed to evaluate the gait dynamics. On the 28th day after SCDEs treatment, the CatWalk system was used to test the rats in each group. The gait parameters were automatically calculated by the analysis software (CatWalk XT 10.6). This experiment mainly evaluated the effect of the following gait parameters on related behavioral changes after SCI, including regularity index, print position, and stances of the hindlimb. An electrophysiological device (YRKJ-G2008; Zhuhai Yiruikeji Co, Ltd, Guangdong, China) was used to analyze motor evoked potential (MEP) in rats four weeks after SCI to evaluate the recovery of nerve conduction function. The latency and amplitude of MEP were recorded and analyzed in the experiment.

### Western blot

The protein concentration was measured by using the BCA assay (Thermo Scientific, #23225, MA, USA). Target proteins were separated by 12% SDS-PAGE and then transferred to a PVDF membrane. After being blocked with 5% nonfat milk in TBST, the PVDF membrane was incubated with the primary antibody overnight and then incubated with the secondary antibody. Protein bands were visualized by using ECL reagents (Thermo Scientific, #35050, MA, USA) and exposed by using the ChemiDoc XRS System (BioRad, USA). Quantitative analysis was performed with Image J software.

The antibodies were listed as follows: rabbit anti-CD9 (1:1000; Cell Signaling Technology, #98327, USA), rabbit anti-Alix (1:1000, Cell Signaling Technology, #92880, USA), rabbit anti-CD63 (1:200; Santa Cruz,#sc-5275, USA), rabbit anti Cleaved Caspase-3 (1:1000; Cell Signaling Technology, #9661, USA), mouse anti-Bcl-2 (1:1000; Abcam, #196495, USA), rabbit anti-iNOS (1:1000; Abcam, #178945, MA, USA), rabbit anti-mannose receptor (1:1000; Abcam, #300621, MA, USA), mouse anti-MFG-E8 (1:500, Santa Cruz, # sc-271574, USA), rabbit anti-SOCS3 (1:1000; Abcam, #280884, MA, USA), rabbit anti-p-STAT3 (1:1000; Cell Signaling Technology, #9145, USA), rabbit anti-STAT3 (1:1000; Cell Signaling Technology, #12640, USA), rabbit anti-glyceraldehyde 3-phosphate dehydrogenase (GAPDH) (1:1000; Abcam, #181602, MA, USA), mouse anti-vinculin (1:10000, Proteintech, #66305-1-Ig), anti-rabbit IgG, HRP-linked Antibody (1:1000; Cell Signaling Technology, #7074, USA), anti-mouse IgG, HRP-linked Antibody (1:1000; Cell Signaling Technology, #7076, USA).

### Immunofluorescence staining

Concerning immunofluorescence staining of cells: cells were fixed, penetrated, and blocked. Then they were incubated with primary antibody at 4 °C overnight and with secondary antibody. PC12 cells were incubated with TUNEL reacting mixture from the TUNEL apoptosis detection kit (Roche, Germany) for 1 h. Finally, the cells were observed with the ultra-high resolution laser confocal microscope (ZEISS LSM 900, Germany). Concerning immunofluorescence staining of spinal cord tissue, the spinal cord segment containing the injury site was taken and fixed with 4% paraformaldehyde overnight. The spinal cord tissues were dehydrated in 30% sucrose solution for 3 days, embedded with OCT, and cut into 10-µm thick slices. The frozen spinal cord sections were permeated and blocked. The tissue sections were incubated with primary antibodies and secondary antibodies. Finally, the tissue sections were observed with the ultra-high resolution laser confocal microscope (ZEISS LSM 900, Germany).

The used primary/second antibodies are listed as follows: rabbit anti-iNOS (1:200; Abcam, #178945 MA, USA), rabbit anti-mannose receptor (1:100; Abcam,#64693, MA, USA), rat anti-F4/80 (1:400; Abcam, #300421, MA, USA), mouse anti-GFAP (1:200; Cell Signaling Technology, 3670, USA), rabbit anti-CD68 (1:200; Abcam, #125212, MA, USA), mouse anti-CD68/ED1 (1:200; Abcam, #31630, MA, USA), rabbit anti-Iba-1 (1:200; Abcam, #178846, MA, USA), mouse anti-MFG-E8 (1:100, Santa Cruz, #sc-271574, USA), rabbit anti-neurofilament heavy polypeptide (1:200; Abcam, #8135 MA, USA), rabbit anti-choline acetyltransferase (1:200; Abcam, #181023, MA, USA), goat anti-rabbit IgG H&L Alexa Fluor 488 (1:400, Abcam, #150077, MA, USA), goat anti-rabbit IgG H&L Cy3 (1:400, Abcam, #6939, MA, USA), goat anti-mouse IgG H&L Alexa Fluor 488 (1:400; Abcam, #150113, MA, USA), and goat anti-mouse IgG H&L Cy3 (1:400; Abcam, #97025, MA, USA).

### Flow cytometry

The treated BMDMs cells were filtered through a 70-μm cell strainer to obtain a single cell suspension, then were incubated with FITC anti-rat CD11b antibody (Biolegend, #201805, USA) and PE anti-rat CD86 antibody (Biolegend, #200308, USA) for 30 min. Cells were permeabilized by Cyto-Fast™ Fix/Perm Buffer Set (Biolenged, #426803, USA) for 15 min. Subsequently, cells were incubated with CD206 (Mannose Receptor) antibody (Santa Cruz, # sc-58986, USA) for 30 min at room temperature in the dark and were incubated with goat anti-mouse IgG H&L (Alexa Fluor® 647/APC) (Abcam, #150115, MA, USA). BD LSRFortessa flow cytometer (BD Biosciences, US) was used for sample collection, and FlowJo was used for data analysis.

The treated PC12 cells were operated according to the staining instructions of FITC Annexin V apoptosis detection kit I (BD Biosciences, #556547, USA), and the cell apoptosis rate was detected immediately by a BD FACSVerse analytical flow cytometer (BD Biosciences, US) after staining, and data analysis was performed by using FlowJo.

### Statistical analysis

GraphPad Prism 9.2.0 software (GraphPad Software, SanDiego, CA, USA) was used for statistical analysis. Data analysis between different groups was carried out via Student’s t-test and one-way analysis of variance (ANOVA), which were followed by Tukey multiple comparison post hoc test. The level of significant difference between groups was defined as: *p < 0.05, **p < 0.01, ***p < 0.001, ****p < 0.0001. Each experiment was repeated at least three times, and the results were shown as the standard error ± mean (SEM).

## Supplementary information

All Western blot images

Checklist

## Data Availability

The authors confirm that the data supporting the conclusions in the paper are presented in the article and its Supplementary Material. Additional data related to this paper are available from the corresponding author.

## References

[1] GBD 2016 Traumatic Brain Injury and Spinal Cord Injury Collaborators. (2019). Global, regional, and national burden of traumatic brain injury and spinal cord injury, 1990-2016: a systematic analysis for the Global Burden of Disease Study 2016. Lancet Neurol.

[2] Huang H, Chen L, Moviglia G, Sharma A, Al Zoubi ZM, He XJ (2022). Advances and prospects of cell therapy for spinal cord injury patients. J Neurorestoratology.

[3] Park J, Decker JT, Margul DJ, Smith DR, Cummings BJ, Anderson AJ (2018). Local immunomodulation with anti-inflammatory cytokine-encoding lentivirus enhances functional recovery after spinal cord injury. Mol Ther.

[4] Kobashi S, Terashima T, Katagi M, Nakae Y, Okano J, Suzuki Y (2020). Transplantation of M2-deviated microglia promotes recovery of motor function after spinal cord injury in mice. Mol Ther.

[5] Ma SF, Chen YJ, Zhang JX, Shen L, Wang R, Zhou JS (2015). Adoptive transfer of M2 macrophages promotes locomotor recovery in adult rats after spinal cord injury. Brain Behav Immun.

[6] Kigerl KA, Gensel JC, Ankeny DP, Alexander JK, Donnelly DJ, Popovich PG (2009). Identification of two distinct macrophage subsets with divergent effects causing either neurotoxicity or regeneration in the injured mouse spinal cord. J Neurosci.

[7] Mosser DM, Edwards JP (2008). Exploring the full spectrum of macrophage activation [published correction appears in Nat Rev Immunol. 2010;10(6):460]. Nat Rev Immunol.

[8] David S, Kroner A (2011). Repertoire of microglial and macrophage responses after spinal cord injury. Nat Rev Neurosci.

[9] Gensel JC, Donnelly DJ, Popovich PG (2011). Spinal cord injury therapies in humans: an overview of current clinical trials and their potential effects on intrinsic CNS macrophages. Expert Opin Ther Targets.

[10] Gensel JC, Zhang B (2015). Macrophage activation and its role in repair and pathology after spinal cord injury. Brain Res.

[11] Kalluri R, LeBleu VS (2020). The biology, function, and biomedical applications of exosomes. Science.

[12] Tkach M, Théry C (2016). Communication by extracellular vesicles: where we are and where we need to go. Cell.

[13] EL Andaloussi S, Mäger I, Breakefield XO, Wood MJ (2013). Extracellular vesicles: biology and emerging therapeutic opportunities. Nat Rev Drug Discov.

[14] Terstappen GC, Meyer AH, Bell RD, Zhang W (2021). Strategies for delivering therapeutics across the blood-brain barrier. Nat Rev Drug Disco.

[15] Sun GD, Li GQ, Li D, Huang WJ, Zhang RW, Zhang H (2018). hucMSC derived exosomes promote functional recovery in spinal cord injury mice via attenuating inflammation. Mater Sci Eng C Mater Biol Appl.

[16] Jessen KR, Mirsky R, Lloyd AC (2015). Schwann cells: development and role in nerve repair. Cold Spring Harb Perspect Biol.

[17] Woodhoo A, Sahni V, Gilson J, Franklin RJM, Blakemore WF, Mirsky R (2007). Schwann cell precursors: a favourable cell for myelin repair in the Central Nervous System. Brain.

[18] Ching RC, Wiberg M, Kingham PJ (2018). Schwann cell-like differentiated adipose stem cells promote neurite outgrowth via secreted exosomes and RNA transfer. Stem Cell Res Ther.

[19] Wong FC, Ye L, Demir IE, Kahlert C (2022). Schwann cell-derived exosomes: Janus-faced mediators of regeneration and disease. Glia.

[20] Pan DY, Zhu SB, Zhang WX, Wei ZJ, Yang FH, Guo ZL (2022). Autophagy induced by Schwann cell-derived exosomes promotes recovery after spinal cord injury in rats. Biotechnol Lett.

[21] Pan DY, Li YJ, Yang FH, Lv ZH, Zhu SB, Shao YX (2021). Increasing toll-like receptor 2 on astrocytes induced by Schwann cell-derived exosomes promotes recovery by inhibiting CSPGs deposition after spinal cord injury. J Neuroinflammation.

[22] Ren YF, Liu WM, Zhang L, Zhang J, Bi JB, Wang T (2021). Milk fat globule EGF factor 8 restores mitochondrial function via integrin-medicated activation of the FAK-STAT3 signaling pathway in acute pancreatitis. Clin Transl Med.

[23] Li ED, Noda M, Doi Y, Parajuli B, Kawanokuchi J, Sonobe Y (2012). The neuroprotective effects of milk fat globule-EGF factor 8 against oligomeric amyloid β toxicity. J Neuroinflammation.

[24] Cheyuo C, Aziz M, Wang P (2019). Neurogenesis in neurodegenerative diseases: role of MFG-E8. Front Neurosci.

[25] Deroide N, Li X, Lerouet D, Vré EV, Baker L, Harrison J (2013). MFGE8 inhibits inflammasome-induced IL-1β production and limits postischemic cerebral injury. J Clin Invest.

[26] Liu F, Chen YJ, Hu Q, Li B, Tang JJ, He Y (2015). MFGE8/Integrin β3 pathway alleviates apoptosis and inflammation in early brain injury after subarachnoid hemorrhage in rats. Exp Neurol.

[27] Shi XL, Cai XY, Di W, Li J, Xu XT, Zhang AW (2017). MFG-E8 selectively inhibited Aβ-induced microglial M1 polarization via NF-κB and PI3K-Akt pathways. Mol Neurobiol.

[28] Wu J, Yang HC, Cheng JJ, Zhang L, Ke YL, Zhu Y (2020). Knockdown of milk-fat globule EGF factor-8 suppresses glioma progression in GL261 glioma cells by repressing microglial M2 polarization. J Cell Physiol.

[29] Bahram Sangani N, Gomes AR, Curfs LMG, Reutelingsperger CP (2021). The role of extracellular vesicles during CNS development. Prog Neurobiol.

[30] Nagatomi J, DeMiguel F, Torimoto K, Chancellor MB, Getzenberg RH, Sacks MS (2005). Early molecular-level changes in rat bladder wall tissue following spinal cord injury. Biochem Biophys Res Commun.

[31] Ozsoy O, Ozsoy U, Stein G, Skouras E, Schempf G, Wellmann K (2012). Functional deficits and morphological changes in the neurogenic bladder match the severity of spinal cord compression. Restor Neurol Neurosci.

[32] Karnup SV, de Groat WC (2020). Propriospinal neurons of L3-L4 segments involved in control of the rat external Urethral Sphincter. Neuroscience.

[33] Wada N, Karnup S, Kadekawa K, Shimizu N, Kwon J, Shimizu T (2022). Current knowledge and novel frontiers in lower urinary tract dysfunction after spinal cord injury: basic research perspectives. Urol Sci.

[34] Kwon J, Lee EJ, Cho HJ, Jang JA, Han MS, Kwak E (2021). Antifibrosis treatment by inhibition of VEGF, FGF, and PDGF receptors improves bladder wall remodeling and detrusor overactivity in association with modulation of C-fiber afferent activity in mice with spinal cord injury. Neurourol Urodyn.

[35] DeFinis JH, Weinberger J, Hou S. Delivery of the 5-HT2A receptor agonist, DOI, enhances activity of the Sphincter muscle during the Micturition reflex in rats after spinal cord injury. Biology. 2021;10:68. Published 2021 Jan 19. Koozekanani SH, Vise WM, Hashemi RM, McGhee RB. Possible mechanisms for observed pathophysiological variability in experimental spinal cord injury by the method of Allen. J Neurosurg. 1976;44:429–434. 10.3171/jns.1976.44.4.0429 1255233

[36] Shang Z, Jia C, Yan H, Cui B, Wu J, W Q (2019). Injecting RNA interference lentiviruses targeting the muscarinic 3 receptor gene into the bladder wall inhibits neurogenic detrusor overactivity in rats with spinal cord injury. Neurourol Urodyn.

[37] Matsumoto Y, Miyazato M, Yokoyama H, Kita M, Hirao Y, Chancellor MB (2012). Role of M2 and M3 muscarinic acetylcholine receptor subtypes in activation of bladder afferent pathways in spinal cord injured rats. Urology.

[38] Guo YX, Ji X, Liu J, Fan DD, Zhou QB, Chen C (2019). Effects of exosomes on pre-metastatic niche formation in tumors. Mol Cancer.

[39] Chen XY, Liu BL, Li XZ, An TT, Zhou Y, Li G (2021). Identification of anti-inflammatory vesicle-like nanoparticles in honey. J Extracell Vesicles.

[40] Kim H, Wang SY, Kwak G, Yang Y, Kwon IC, Kim SH (2019). Exosome-guided phenotypic switch of M1 to M2 macrophages for cutaneous wound healing. Adv Sci.

[41] Ni YQ, Zhan JK, Liu YS (2020). Roles and mechanisms of MFG-E8 in vascular aging-related diseases. Ageing Res Rev.

[42] Cheyuo C, Jacob A, Wu R, Zhou M, Qi L, Dong WF (2012). Recombinant human MFG-E8 attenuates cerebral ischemic injury: its role in anti-inflammation and anti-apoptosis. Neuropharmacology.

[43] Segura E, Nicco C, Lombard B, Véron P, Raposo G, Batteux F (2005). ICAM-1 on exosomes from mature dendritic cells is critical for efficient naive T-cell priming. Blood.

[44] Rong Y, Ji C, Wang Z, Ge X, Wang J, Ye W (2021). Small extracellular vesicles encapsulating CCL2 from activated astrocytes induce microglial activation and neuronal apoptosis after traumatic spinal cord injury. J Neuroinflammation.

[45] Fan H, Zhang K, Shan L, Kuang F, Chen K, Zhu K (2016). Reactive astrocytes undergo M1 microglia/macrohpages-induced necroptosis in spinal cord injury. Mol Neurodegener.

[46] Milich LM, Ryan CB, Lee JK (2019). Correction to: The origin, fate, and contribution of macrophages to spinal cord injury pathology. Acta Neuropathol.

[47] Gao YY, Tao T, Wu D, Wu D, Zhuang Z, Lu Y (2021). MFG-E8 attenuates inflammation in subarachnoid hemorrhage by driving microglial M2 polarization. Exp Neurol.

[48] Yao X, Zhang Y, Hao J, Duan HQ, Zhao CX, Sun C, et al. Deferoxamine promotes recovery of traumatic spinal cord injury by inhibiting ferroptosis [published correction appears in Neural Regen Res. 2019;14:532–41. Neural Regen Res.2019;14:1068. 10.4103/1673-5374.245480 PMC6334606 30539824

