# Supplementary figures and images for: Schwann cell-derived exosomes containing MFG-E8 modify macrophage/microglial polarization for attenuating inflammation via the SOCS3/STAT3 pathway after spinal cord injury
Source: Cell Death Dis. 2023 Jan 30;14(1):70. doi: 10.1038/s41419-023-05607-4 (PMC9887051; doi:10.1038/s41419-023-05607-4)

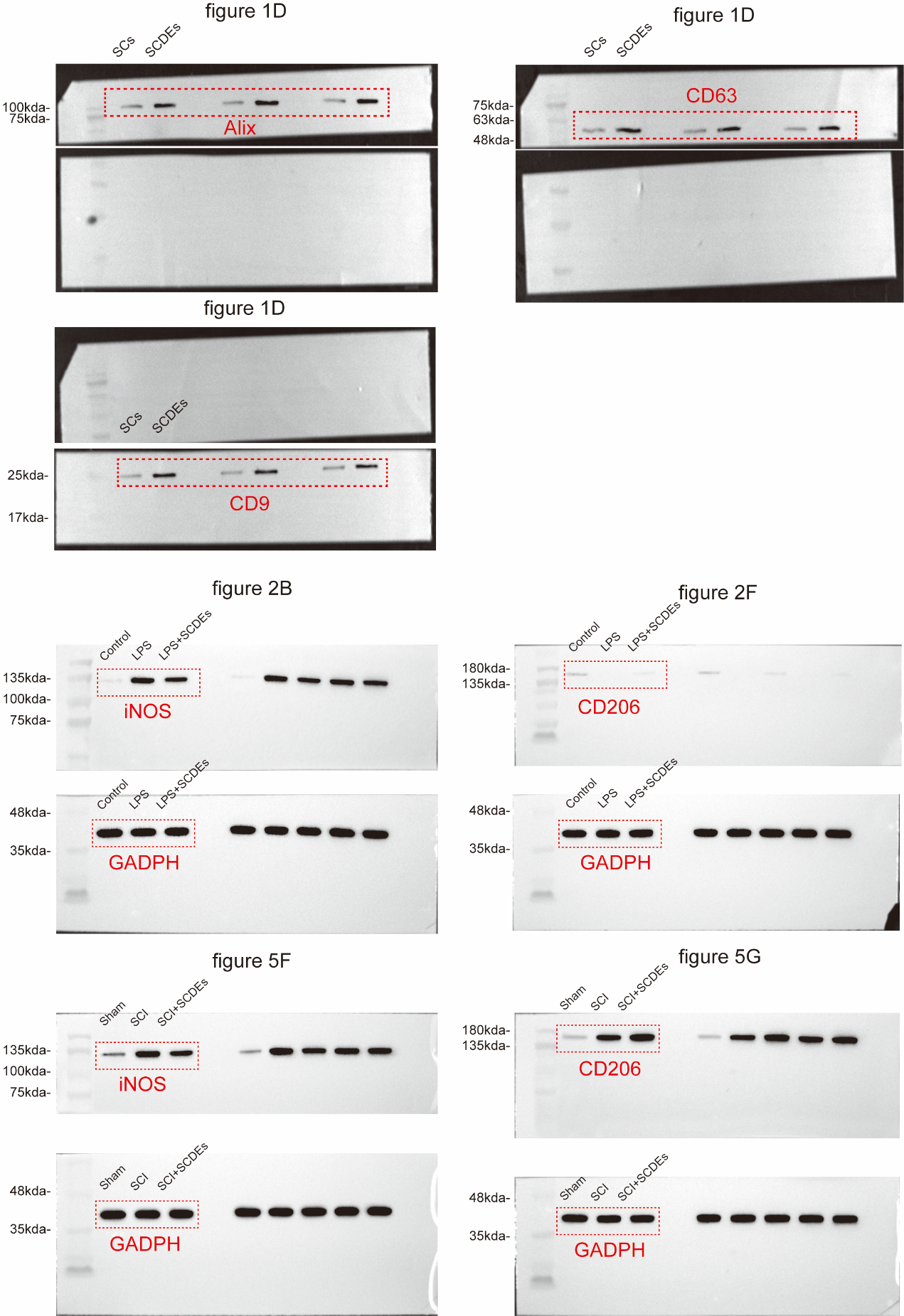


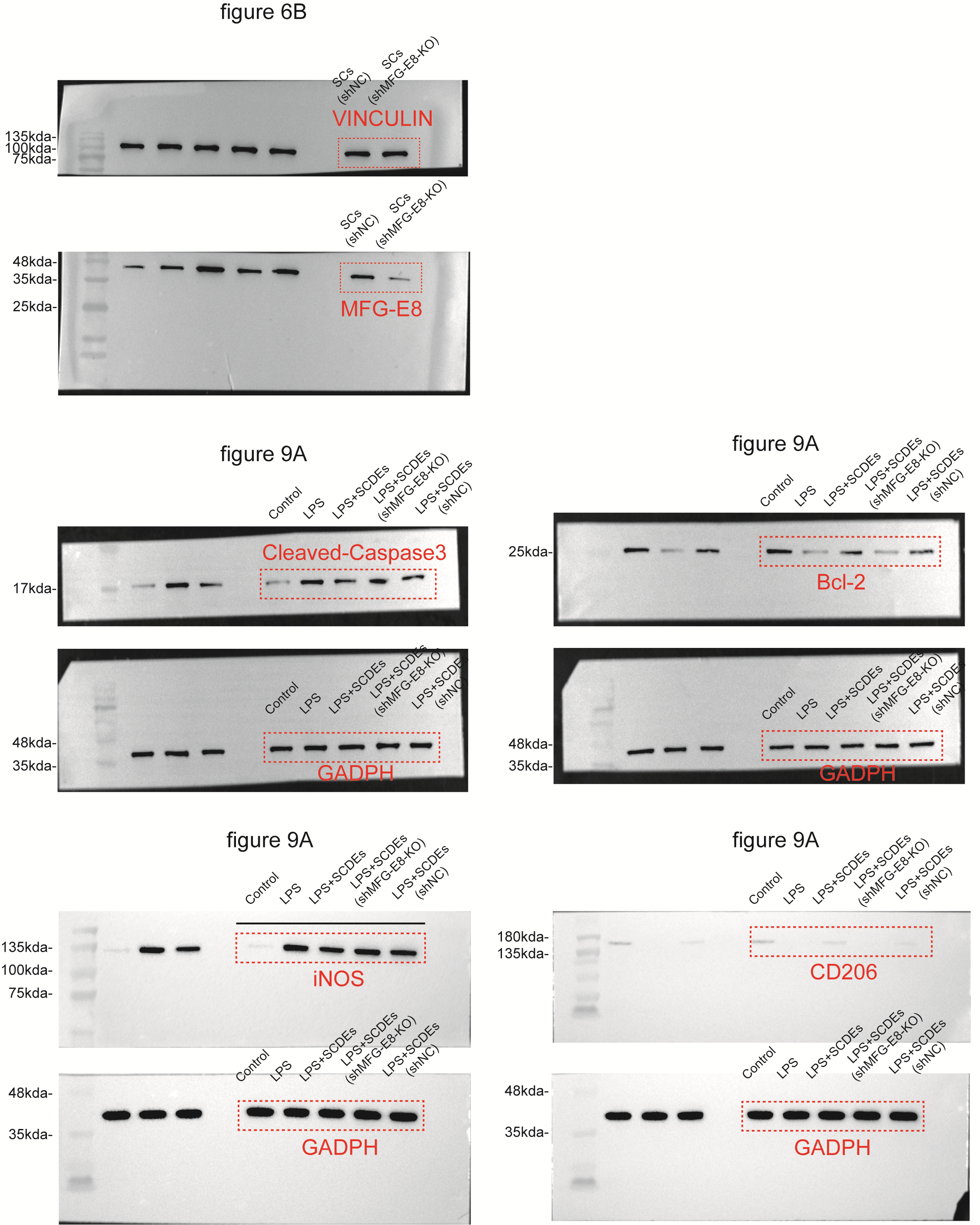

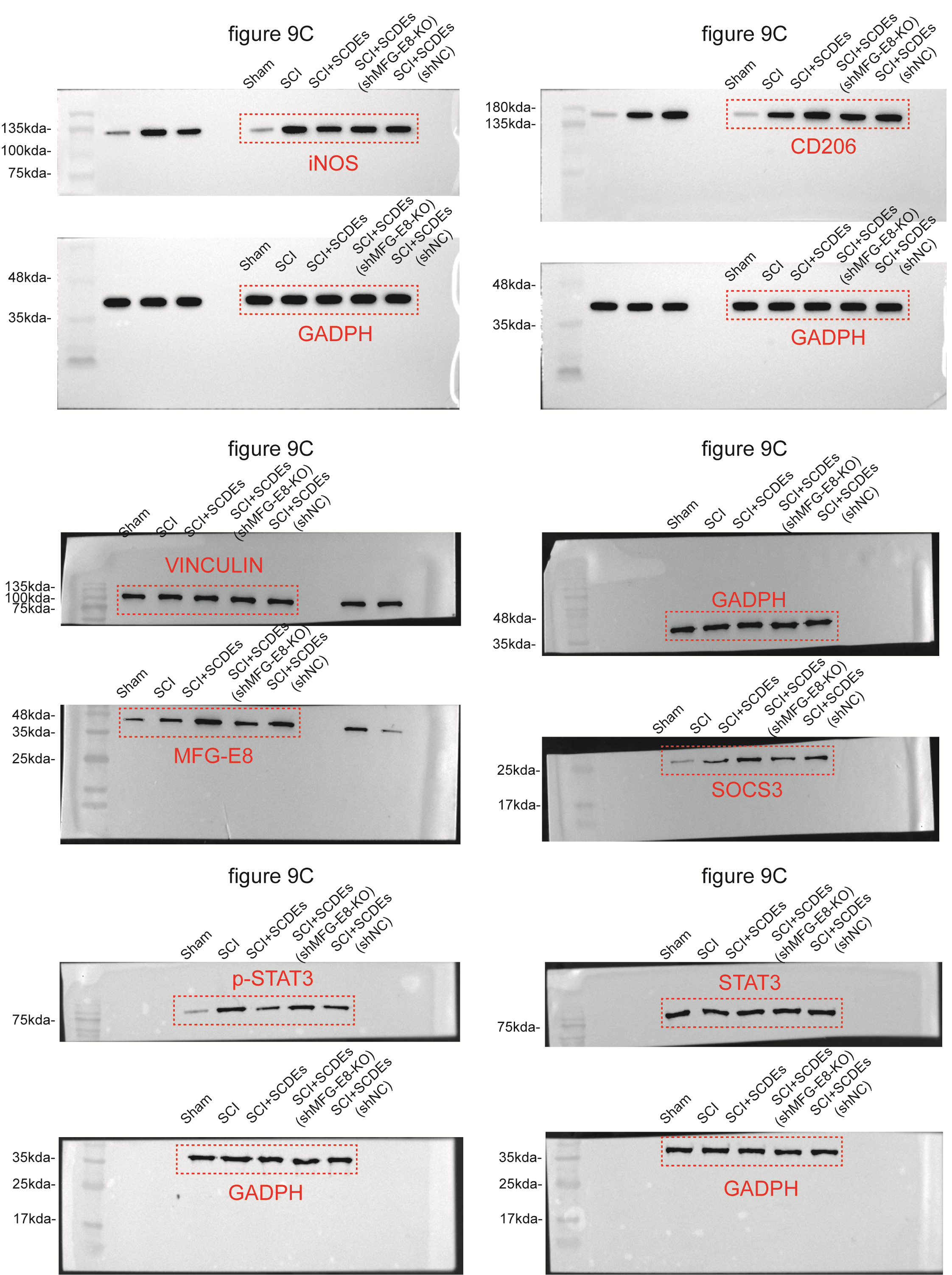

Supplement: Supplementary file 1 — All Western blot images [file 41419_2023_5607_MOESM1_ESM.docx]
